# Quality control for terms and definitions in ontologies and taxonomies

**DOI:** 10.1186/1471-2105-7-212

**Published:** 2006-04-19

**Authors:** Jacob Köhler, Katherine Munn, Alexander Rüegg, Andre Skusa, Barry Smith

**Affiliations:** 1 Biomathematics and Bioinformatics Division, Rothamsted Research, Harpenden, UK; 2 Department of Philosophy, University at Buffalo, NY, USA; 3 Institute for Formal Ontology and Medical Information Science, Saarland University, Saarbrücken, Germany; 4 Technical Faculty, Bielefeld University, Bielefeld, Germany

## Abstract

**Background:**

Ontologies and taxonomies are among the most important computational resources for molecular biology and bioinformatics. A series of recent papers has shown that the Gene Ontology (GO), the most prominent taxonomic resource in these fields, is marked by flaws of certain characteristic types, which flow from a failure to address basic ontological principles. As yet, no methods have been proposed which would allow ontology curators to pinpoint flawed terms or definitions in ontologies in a systematic way.

**Results:**

We present computational methods that automatically identify terms and definitions which are defined in a circular or unintelligible way. We further demonstrate the potential of these methods by applying them to isolate a subset of 6001 problematic GO terms. By automatically aligning GO with other ontologies and taxonomies we were able to propose alternative synonyms and definitions for some of these problematic terms. This allows us to demonstrate that these other resources do not contain definitions superior to those supplied by GO.

**Conclusion:**

Our methods provide reliable indications of the quality of terms and definitions in ontologies and taxonomies. Further, they are well suited to assist ontology curators in drawing their attention to those terms that are ill-defined. We have further shown the limitations of ontology mapping and alignment in assisting ontology curators in rectifying problems, thus pointing to the need for manual curation.

## Background

Taxonomies and ontologies are of increasing importance in functional genomics and molecular biology, and the Gene Ontology [1] has established itself as one of the most important computational resources in these and related fields. Several of the ontologies in the Open Biomedical Ontologies (OBO) Consortium, of which GO is the best known resource, have had a major impact on the annotation of genomes [2] and are also often used as controlled vocabularies in database integration systems [3]. Applications are increasingly exploiting ontologies like GO for such tasks as microarray analysis [4,5], text mining [6], database integration [7], and measurement of the semantic similarity of terms used in annotations [8]. As discussed in [9-18], when ontologies are built following certain well-established design principles principles, it is possible for applications to take advantage of their data structure. Our investigation here pertains to the ways GO and similar ontologies fall short of conforming to principles that apply to the naming and definitions of ontological terms. Since ontologies need to be used by diverse groups, human intelligibility is absolutely crucial. We note with satisfaction that the GO Consortium has recognized the importance of the problems addressed in this communication, and is taking steps to rectify them in conjunction with the developers of other OBO Ontologies. The proposals advanced in [19] are also being applied in on-going revisions of GO's definitions.

We will use the terms 'controlled vocabulary', 'taxonomy' and 'ontology' according to their definitions in [20], without claiming that this is the only way to define them. We will thus consider a *controlled vocabulary *to be a set of *nodes *each of which is associated with an *identifier, term, definition*, and an optional set of *synonyms*. In *ontologies *the nodes are linked by directed edges, thus forming a graph. This graph represents a counterpart structure on the side of entities (classes, universals) in reality, and its edges represent the relations (e.g. *is-a *or *part-of*) which hold between these entities. If a node has a parent node in the *is-a *hierarchy, then we say that the corresponding class is *subsumed *by this parent node.

Whereas this publication presents methods for assessing the quality of names and definitions of terms in ontologies and taxonomies, there are of course several other methods for assessing different aspects of the quality of ontologies. Several research programs [21,22] use both computational methods and manual ontology curation in order to overcome shortcomings in GO; we ourselves have already pointed to a variety of such shortcomings and have suggested possible ways to overcome them [16,17,23]. Computational methods exist for assessing the quality of certain other aspects of ontologies. Ontologies that are represented using Description Logic based languages such as OWL, allow the definition of constraints, assertions and other suitable data structures, which can be used for consistency and quality checking at the schema and the entry level [24-28], as well as for removing redundancy [29]. These methods also allow the assessment of features of ontologies relevant to human usability and suitability for a specific application [30]. However, these methods are not suitable to assess the quality of those free text definitions, names and synonyms which are the primary "handles" for human users. Standard readability scores such as the Fog Index or the Flesh reading easy formula [31] are commonly used as indicators to assess how easy it is to understand a given text. These scores rely on measures such as the average length of sentences, number of punctuation marks and the percentage of words which occur in an "easy word" list. Such readability scores should normally be applied to texts which are at least 200 words long. Since definitions in most OBO ontologies are 10 words long or less, the applicability of the readability scores to definitions is questionable. However, there are other more important criteria for assesing the quality of a definition which are not covered by the readability scores. According to [32], the following five rules are recommended for the formulation of good definitions: 1.) Focus on essential features. 2.) Avoid circularity, 3.) Capture the correct extension, 4.) Avoid figurative or obscure language and 5.) Be affirmative rather than negative. These rules are based on the principles of Aristotelian definitions, which are also the basis for the principles that are applied to definitions in ontologies such as the FMA (Foundational Model of Anatomy)[14]. According to [16], especially two of these five characteristics from [32] are suitable to mark a definition as well structured, namely *avoidance of circularity *and *intelligibility*:

Rule 2: *Avoid circularity*. Since a circular definition uses the term being defined as part of its own definition, it can't provide any useful information; either the audience already understands the meaning of the term, or it cannot understand the explanation that includes that term. Thus, for example, there isn't much point in defining "cordless 'phone" as "a telephone that has no cord."

Rule 4: *Avoid figurative or obscure language*. Since the point of a definition is to explain the meaning of a term to someone who is unfamiliar with its proper application, the use of language that doesn't help such a person learn how to apply the term is pointless. Thus, "happiness is a warm puppy" may be a lovely thought, but it is a lousy definition.

Here we propose and evaluate computational methods which are suitable to assess these two main criteria for a good definition. We will use the term "intelligibility" in the following when we refer to the rule concerning avoidance of figurative or obscure language. This is because in the example domain at issue it is primarily the amount of technical terminology used that is of concern.

The importance of defining ontological terms in a noncircular and intelligible way should be clear when we consider the main role of ontologies like the GO in biology and bioinformatics, which is to facilitate genome annotation. Biologists use terms from ontologies to define the specific roles of genes in a way that is concise yet unambiguous. However, when classes lack clear definitions, it is easy for curators who annotate genomes, as well as for experimental biologists who rely on these annotations, to make mistakes. Experimental biologists may be misled by misannotations, or they may misunderstand the significance of a correct annotation if the latter lacks a meaningful definition. Jane Lomax (GO curation coordinator, EBI) asserts that "there have been many occasions where wrong annotations have arisen from dodgy definitions" (personal communication). The GO consortium is fully aware of the importance of providing high quality definitions and states in the GO Editorial Guide [33] "Always define new terms: If you create a new term, or refine a term, you should add a definition for it, and note the references used in composing the definition (...). Write definitions carefully: Definitions should explain clearly to the reader what is meant by a particular term. They should be concise, full sentences.". Clearly the GO team is fully aware of quality issues, including the provision of high quality definitions, even though they recognize also that providing high-quality definitions for all terms is a challenging and time-consuming task.

This paper is a contribution to the methodology of bioinformatics, and its main results are the methods we developed for identifying circular and unintelligible terms/definitions in ontologies and taxonomies. To demonstrate their usability, we applied them to the Gene Ontology, and asked domain experts to manually assess the circularity and intelligibility of a subset of the terms which we scored. These methods are generic in nature, i.e. they can be incorporated into existing ontology editors where they would have a very direct impact on the quality of the names and definitions used, while curators could directly use these scores to identify potentially flawed terms that require improved definitions.

The methods presented in this publication are applicable to any ontology or taxonomy. We selected GO to demonstrate the potential of these methods because it is one of the most mature ontological resources in the biomedical domain and has benefited from significant financial and human investment over a long period of time, as well as from substantial feedback and contributions from the scientific community. It is thus very likely that most other ontologies and taxonomies contain at least as many ill-defined concepts as there are in GO. Because GO is subject to a permanent process of curation, some of the problems we present here have been rectified in more recent versions. The version of GO which contains the examples we present in this publication can be retrieved from GO's sourceforge repository (revision 2.1707, February 2004).

## Results and discussion

In this section, we present three main results: An index for automatically assessing circularity, an index for automatically assessing intelligibility of terms and definitions, and a use case in which the performance of these indexes is demonstrated in application to the Gene Ontology. At the end of the section we discuss how definitions can be rewritten in a more intelligible and non-circular way.

### Circularity Index

Consider:

**id: **GO:0042270

**term: **Protection from natural killer cell mediated cytolysis

**definition: **The process of protecting a cell from cytolysis by natural killer cells.

This is an example of a circular definition which illustrates also how a definition may be circular even though its component words differ syntactically in several respects from the words used in the term defined. They may differ in flexion (declension and conjugation), form (singular versus plural), or capitalization; and they may also contain stopwords such as "the", "of" "a", "from". From a semantic point of view, however, such differences contribute little to the definition. In our example, the only words in the definition that differ semantically from those in the term defined are "process" and "mediated". But even "process" is not informative, since it appears in the root term of GO's molecular process ontology, so that GO's hierarchical structure already reflects the fact that the entity in question is a process.

We measured the degree of circularity of a definition by counting those words occurring in both the definition and the term and relating this number to the number of words in the definition. Words that appear twice in the definition, even if in different forms (singular or plural) are only counted once. Thus we define the circularity index *C *as follows:

C:=|s(def\stop)∩s((term∪syns)\stop)||s(def\stop)|
 MathType@MTEF@5@5@+=feaafiart1ev1aaatCvAUfKttLearuWrP9MDH5MBPbIqV92AaeXatLxBI9gBaebbnrfifHhDYfgasaacH8akY=wiFfYdH8Gipec8Eeeu0xXdbba9frFj0=OqFfea0dXdd9vqai=hGuQ8kuc9pgc9s8qqaq=dirpe0xb9q8qiLsFr0=vr0=vr0dc8meaabaqaciaacaGaaeqabaqabeGadaaakeaacqWGdbWqcqGG6aGocqGH9aqpdaWcaaqaamaaemaabaGaem4CamNaeiikaGIaemizaqMaemyzauMaemOzayMaeiixaWLaem4CamNaemiDaqNaem4Ba8MaemiCaaNaeiykaKIaeyykICSaem4CamNaeiikaGIaeiikaGIaemiDaqNaemyzauMaemOCaiNaemyBa0MaeyOkIGSaem4CamNaemyEaKNaemOBa4Maem4CamNaeiykaKIaeiixaWLaem4CamNaemiDaqNaem4Ba8MaemiCaaNaeiykaKcacaGLhWUaayjcSdaabaWaaqWaaeaacqWGZbWCcqGGOaakcqWGKbazcqWGLbqzcqWGMbGzcqGGCbaxcqWGZbWCcqWG0baDcqWGVbWBcqWGWbaCcqGGPaqkaiaawEa7caGLiWoaaaaaaa@6C78@

where

*s *= the function that returns the set of all distinct lower case converted word stems from a set of words

*def *= the set of all words used in the definition

*term *= the set of all words used in the term

*syns *= the set of all words used in the synonyms of the term

*stop *= the set of stopwords

When applied to the abovementioned term 'protection from natural killer cell mediated cytolysis' the formula yields a circularity index of 0.833. The non-circular definition:

**id: **GO:0050919

**term: **negative chemotaxis

**definition: **The directed movement of a motile cell or organism towards a lower concentration in a concentration gradient of a specific chemical

in contrast, has a circularity index of 0, reflecting the fact that the definition and the term contain no words in common.

The index compares the information contained in the term to the information contained in the definition but it does this in such a way as to take synonyms into account. Thus for example the term

**id: **GO:0005105

**term: **breathless binding

**synononyms: **breathless ligand, FGFR1 binding, FGFR1 ligand, type 1 fibroblast growth factor receptor ligand, type 1 fibroblast growth factor receptor binding

**definition: **Interacting selectively with the type 1 fibroblast growth factor receptor (FGFR1)

has 5 synonyms, and 7 out of 9 non-stopwords in the definition also occur in at least one of the synonyms. Although this definition is an improvement over a mere list of names, it still does little more than reiterate the information contained in the term and its synonyms. In consequence, the circularity index of this term is relatively high (0.778). An example for a term with a circularity index of 0.5 is:

**id: **GO:0050948

**term: **positive regulation of early stripe melanocyte differentiation

**definition: **Any process that activates or increases the rate of early stripe melanocyte differentiation.

Ontologies such as the FMA [14] aim at avoiding circularity completely. To identify terms and definitions that do not meet their quality standards, one would apply a threshold of *C *≥ 0.

### Intelligibility index

A system of definitions should identify a small number of primitives, such as 'process' or 'component', which are as far as possible intelligible in their own right. Apart from these, every term in the system should have a definition which meets basic standards of adequacy [34]. It is to this end that we introduce an index that can be used to quantify the *intelligibility *of both definitions and of terms defined.

Consider:

**id: **GO:0050566

**term: **asparaginyl-tRNA synthase (glutamine-hydrolyzing) activity

**definition: **Catalysis Cyc:6.3.5.6-RXN,

We believe that to most GO users neither the definition nor the term given here is self-explanatory. Rather, their understanding requires background knowledge drawn from a highly specialized biological sub-discipline. We question also whether terms and definitions of this sort are in any sense intelligible to computers programmed for automatic information extraction. Actually, this GO term existed only for a short time in the GO. It is a case where both the term and the definition have been imported from the MetaCyc database [35]. Soon after it was imported, the GO team became aware of this flawed term and corrected it.

To isolate cases marked by low intelligibility we counted how many of the words occurring in a given GO definition are defined as terms in WordNet [36], a lexical reference system that has basically the same underlying data structure as OBO ontologies, but with a much broader coverage. WordNet was suitable to this task because it contains a number of commonly used words, including technical words drawn from biomedical terminology, but they are terms whose level of technicality does not exceed that which a broad base of biologists and biomedical researchers can be expected to have mastered. Its domain thus covers most areas of the common language used by both scientists who are specialists in a given field and those who are not. We define the intelligibility index of a definition in an ontology or taxonomy as follows:

Idef:=|s(def\stop)∩s(wn)||s(def\stop)|
 MathType@MTEF@5@5@+=feaafiart1ev1aaatCvAUfKttLearuWrP9MDH5MBPbIqV92AaeXatLxBI9gBaebbnrfifHhDYfgasaacH8akY=wiFfYdH8Gipec8Eeeu0xXdbba9frFj0=OqFfea0dXdd9vqai=hGuQ8kuc9pgc9s8qqaq=dirpe0xb9q8qiLsFr0=vr0=vr0dc8meaabaqaciaacaGaaeqabaqabeGadaaakeaacqWGjbqsdaWgaaWcbaGaemizaqMaemyzauMaemOzaygabeaakiabcQda6iabg2da9maalaaabaWaaqWaaeaacqWGZbWCdaqadaqaaiabdsgaKjabdwgaLjabdAgaMjabcYfaCjabdohaZjabdsha0jabd+gaVjabdchaWbGaayjkaiaawMcaaiabgMIihlabdohaZnaabmaabaGaem4DaCNaemOBa4gacaGLOaGaayzkaaaacaGLhWUaayjcSdaabaWaaqWaaeaacqWGZbWCdaqadaqaaiabdsgaKjabdwgaLjabdAgaMjabcYfaCjabdohaZjabdsha0jabd+gaVjabdchaWbGaayjkaiaawMcaaaGaay5bSlaawIa7aaaaaaa@5D80@

Here,

*s *= the function that returns the set of all distinct lower case word stems from a set of words

*def *= the set of all words used in the definition

*term *= the set of all words used in the term

*stop *= the set of stopwords

*wn *= all words defined in WordNet

We can also determine the Intelligibility Index of a term, *I*_*term*_, by replacing *def *with *term *as follows:

Iterm:=|s(term\stop)∩s(wn)||s(term\stop)|
 MathType@MTEF@5@5@+=feaafiart1ev1aaatCvAUfKttLearuWrP9MDH5MBPbIqV92AaeXatLxBI9gBaebbnrfifHhDYfgasaacH8akY=wiFfYdH8Gipec8Eeeu0xXdbba9frFj0=OqFfea0dXdd9vqai=hGuQ8kuc9pgc9s8qqaq=dirpe0xb9q8qiLsFr0=vr0=vr0dc8meaabaqaciaacaGaaeqabaqabeGadaaakeaacqWGjbqsdaWgaaWcbaGaemiDaqNaemyzauMaemOCaiNaemyBa0gabeaakiabcQda6iabg2da9maalaaabaWaaqWaaeaacqWGZbWCdaqadaqaaiabdsha0jabdwgaLjabdkhaYjabd2gaTjabcYfaCjabdohaZjabdsha0jabd+gaVjabdchaWbGaayjkaiaawMcaaiabgMIihlabdohaZnaabmaabaGaem4DaCNaemOBa4gacaGLOaGaayzkaaaacaGLhWUaayjcSdaabaWaaqWaaeaacqWGZbWCdaqadaqaaiabdsha0jabdwgaLjabdkhaYjabd2gaTjabcYfaCjabdohaZjabdsha0jabd+gaVjabdchaWbGaayjkaiaawMcaaaGaay5bSlaawIa7aaaaaaa@6251@

The intelligibility index can take values between 0 (low intelligibility) and 1 (high intelligibility). The example just given has an intelligibility index of 0.25. An example for a term where the definition has an intelligibility index of 0.7 is:

**id: **GO:0046479

**term: **glycosphingolipid catabolism

**definition: **The breakdown into simpler components of glycosphingolipid, a compound with residues of sphingoid and at least one monosaccharide

Whereas this term still relies on some technical terminology, the definition of the following GO term which has an intelligibility index of 1, should also be understandable to non scientists:

**id: **GO:0042600

**term: **chorion

**definition: **A protective, noncellular membrane that surrounds the eggs of various animals including insects and fish.

The intelligibility index reliably spots definitions that contain much technical terminology. But it is worth noting that it does not rule out the case where a given text string is unintelligible even though it uses only familiar words.

### Use case: Gene Ontology

**Figure 1:**
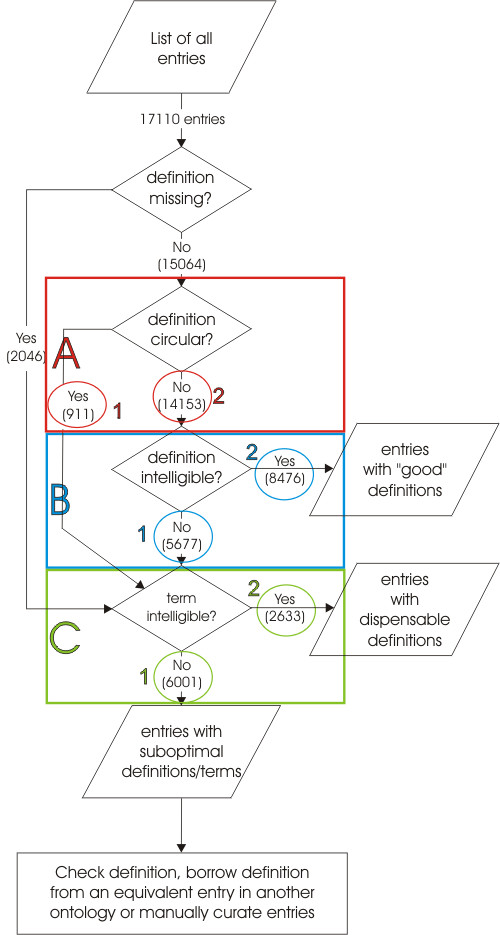
**Workflow for the computational evaluation of the quality of terms and definitions in controlled vocabularies**. Definitions are considered to be circular if they have a circularity index *C *≥ 0.5 (see section "Circularity Index") and as intelligible if they have an intelligibility index *I *≤ 0.7 (see section "Intelligibility Index"). The results of applying this workflow to the Gene Ontology are available in the Additional files: *A circularity of definition: *A1 (see Additional file 1) – Circular definitions (circularity index ≥ 0.5), A2 (see Additional file 2) – Non-circular definitions (circularity index < 0.5) *B intelligibility of definition: *B1 (see additional file 3) – Unintelligible definitions (intelligibility index < 0.7), B2 (see Additional file 4) – Intelligible definitions (intelligibility index ≥ 0.7) *C intelligibility of term: *C1 (see Additional file 5) – Unintelligible terms (intelligibility index < 0.7), C1a (see Additional file 6) – Unintelligible terms with proposed alternative definitions (intelligibility index < 0.7), C2 (see Additional file 7) – Intelligible terms (intelligibility index ≥ 0.7).

We set up a workflow (see Figure 1) designed to draw the attention of ontology curators to ill-defined terms. We then aligned GO terms to equivalent terms in other ontologies, in order to assess the possibility of replacing problematic definitions in GO with definitions borrowed from other ontologies. The results of the use case are provided as tab delimited files (see Additional files 1 – 7) which are related to the different steps of the workflow in Figure 1.

The workflow requires the definition of thresholds. On consideration of the above-mentioned examples, we think that a threshold for circularity of C ≥ 0.5 and a threshold for intelligibility (I_*def *_or I*t*_*erm*_)≤ 0.7 is a good default value. Yet we do not insist on these thresholds for all purposes, and we imagine that the threshold chosen will in practice reflect a compromise between the desire for quality in the ontology and the time which can be spent in rewriting circular terms and definitions. Starting with a high threshold and iteratively decreasing the threshold would allow curators to focus on the most problematic definitions first.

#### Circularity of GO terms

A non-circular definition with an index of 0 indicates that the term and the definition contain no words in common. This was the case for 2,117 GO terms. As measured by the C ≥ 0.5 threshold, 5.32 % of all GO definitions (911 terms) are circular: they are redundant, containing no more information than do the corresponding terms themselves. In other words they perform no service, either for human users or for computers programmed to use GO for tasks of automatic information retrieval.

Intelligibility of definitions of GO terms: We stipulated that those terms and definitions are to be flagged for additional manual curation which have an intelligibility index (I_*def *_or I*t*_*erm*_) ≤ 0.7. This was the case for 5677 GO terms.

Many low-intelligibility terms in GO describe biochemical reactions. The reason for this is that the definitions for such terms employ the names of the corresponding chemical compounds, very few of which are contained in WordNet. It could of course be argued that such names actually are intelligible for a specific audience, and that, even though many biologists will not know the names or formulas of the compounds involved in a given biochemical reaction, the reaction in question is still specified in a way that is at least in principle apprehendable by most biologists. This interpretation at least is the one taken in the Gene Ontology Next Generation Project [21], in which the human- and computer-readable representations of the types of entities involved in metabolism and the linkage of such representations to external ontologies and databases are in fact active fields of research. Therefore, depending on the application scenario, users of the proposed indexes may choose to exclude such terms which are in principle intelligible from the analysis.

#### Intelligibility of names and synonyms of GO terms

It could however be argued that if a term (or one of its synonyms) is intelligible in its own right, then the term itself can serve as its own definition. Thus, we used the intelligibility of the names and synonyms of the terms to narrow down the list of problematic terms. As a result of this step, 6001 ill-defined terms remain out of the 17,110 terms which were included in this particular release.

#### Ontology alignment: can definitions automatically be borrowed from other ontologies and taxonomies?

The application of the workflow depicted in Figure 1 results in the isolation of a subset of 6,001 GO terms that are defined circularly, have an unintelligible definition, or have no definition at all and are also such that the names and synonyms are not intelligible.

The next step of the workflow was to see if it was possible to replace suboptimal or missing definitions with definitions from other ontologies or controlled vocabularies by automatically aligning GO to MeSH [37], WordNet 2.0 [36], and the Enzyme Nomenclature [38]. Of the 6,001 (5,916 non-obsolete) cases in which definitions were found to be circular, missing, or to have a low intelligibility index either for the definition or for the associated term, only 2,831 had an equivalent term in one of the other resources mentioned. Although an equivalent term was found for almost half of the terms, the associated definitions were in most cases no better with respect to circularity or intelligibility than the definitions already existing in GO. This observation is based on the two scores which we introduced and evaluated in this paper, and on the feedback we received when these alternative definitions were shown to our evaluators (see below). This tells us that circular and unintelligible definitions are not only a problem in GO. Thus the rectification of problems in GO and other taxonomies will require manual curation, since only on a case-by-case basis can it be decided whether a definition should be replaced, supplemented, or completely rewritten. In the next section we discuss guidelines for such manual curation.

### Evaluation

We asked three biologists (2 postdocs > 10 years postdoc experience, 1 BSc who graduated about 2 years ago) and a bioinformatician (MSc, recently graduated) to evaluate both for circularity and for intelligibility the fifty highest and the fifty lowest ranking GO terms (= 200 terms in total). The high and low scoring terms were presented in random order, and the scores were not visible to the evaluators. For reasons discussed in section "Intelligibility of definitions of GO terms", we excluded terms describing biochemical reactions from the evaluation. The evaluators were asked to answer the following questions with 'yes' or 'no'.

For Circularity:

Q1: Is the definition not circular, i.e. does the definition provide more information than the term itself?

For Intelligibility two questions were asked:

Q2: Is the definition intelligible, i.e. did you roughly understand the meaning of the GO entry by reading the definition?

Q3: Is the definition intelligible, i.e. are you able to fully understand the meaning of the GO entry without requiring further reading of other sources?

The evaluation results are summarised in Table 1. The full evaluations are available for the evaluation of the circularity index (see Additional file 8) and the evaluation of the intelligibility index (see Additional file 9). In short, the evaluation results gained in response to the three questions show that:

Q1: the circularity scores are in good agreement with the manual assessment of circularity;

Q2: terms which receive a low intelligibility score, are still useful to give users a rough idea of their nature;

Q3: terms which receive a low intelligibility score, do not allow users to fully understand the meaning of an entry without requiring that other sources be consulted.

Regarding intelligibility, it seems that the biologists (but not the bioinformatician) had sufficient background knowledge to understand *in principle *the terms which received low scores in the intelligibility index (Q2), although in many cases even the biological domain experts were not able to fully understand the low scoring terms without referring to external sources (Q3). As already mentioned, the GO Editorial Guide states "Write definitions carefully. Definitions should explain clearly to the reader what is meant by a particular term. They should be concise, full sentences...". Thus it seems that the intelligibility index should be applicable as quality criterion for definitions at least within the framework of the GO. Interestingly, for Q3, one of the Postdocs found only 29 out of 50 terms which received a high intelligible score to actually be fully understandable. When it comes to definitions which received a low intelligibility score, all evaluators agreed that these are not fully understandable. In other words, the intelligibility index picks out in a relatively reliably manner a large number of terms which are not fully intelligible, although it probably cannot identify all unintelligible terms.

The following GO term exemplifies the different results obtained for Q2 and Q3:

**id: **GO:0018070

**term: **peptidyl-serine phosphopantetheinylation

**definition: **The posttranslational phosphopantetheinylation of peptidyl-serine to form peptidyl-O-phosphopantetheine-L-serine.

The definition gives the users a rough idea of the meaning of the term, since they understand that "peptidyl-serine" and "peptidyl-O-phosphopantetheine-L-serine" are chemical compounds, and that the former is converted to the latter by an ominous process called "phosphopantetheinylation". However, in order properly to understand what a gene which is annotated with this GO term does, users would have to look up what these specific compounds do, what chemical structure they have, as well as the exact meaning of "phosphopantetheinylation". According to the feedback we received from the evaluators, the same principles apply to GO terms that describe biochemical reactions, i.e. they are also in principle understandable, although in most cases further reading of external sources is required in order to fully understand the meaning of such GO-terms. Yet, it may well be questioned if a definition in an ontology should require reading of other definitions. Although such a definition may be correct and sufficiently precise, it is of limited use to biologists who often have to go through hundreds of GO terms and definitions at a single sitting when for example gene annotations are used for the interpretation of microarray results.

In summary, the circularity index is well suited to draw the attention of ontology curators to terms which are defined in a circular way. The intelligibility index can be used a) to identify terms which are only understandable to specialised domain experts, but not understandable to the broader scientific community and b) to identify terms which require further reading of external sources to fully understand their meaning.

### Improving definitions

The guidelines for the manual curation required for improving definitions are straightforward. To define terms in a non-circular way, one should avoid reiterating the information that is already inherent in the term itself. Rather, this information should be broken down and its components described individually, ideally according to the rules laid down in [14]. Term names and definitions are often relatively short in ontologies. Therefore, it is not surprising that the relatively small changes to terms and definitions can make a big difference, which is also reflected in the scores that these terms receive.

Definitions with low intelligibility are best addressed by avoiding technical terminology in the definition, or where this is not possible, by adding words that clarify the nature of the technical term (whether it is a substance, a disease, or a specific sort of process, and so forth). This will make the definition more readily accessible to human users, something which will be marked by an increase in the intelligibility index. Definitions should nonetheless not be longer than necessary, in order to preserve the efficiency with which the terminology can be used. A guideline for deciding how long a definition needs to be is to ask whether it defines the term in a way that differentiates it clearly from other related entries.

In the following we will use two examples to illustrate how terms can be improved. First consider a GO term whose definition received the highest possible score of 1 for circularity:

**id: **GO:0001655

**term: **urogenital system development

**definition: **the development of the urogenital system

The latest GO version (Release February 2005) already provides a revised definition for this term which serves as a good example of the sorts of improvements which can be made:

**id: **GO:0001655

**term: **urogenital system development

**definition: **Processes aimed at the progression of the urogenital system over time, from its formation to the mature structure.

An example of a term with low intelligibility (with a score of 0.3) is:

**id: **GO:0006190

**term: **inosine salvage

**definition: **Any process that generates inosine, hypoxanthine riboside, from deriviatives of it without de novo synthesis.

This definition succeeds at precisely defining a biochemical process, yet it fails to indicate its significance against the larger background of a biological system, rendering it opaque to most users. An improved version of this GO term could be written as follows:

**id: **GO:0006190

**term: **inosine salvage

**synonyms: **hypoxanthine riboside salvage

**definition: **Any process that generates inosine, a nucleic acid important for RNA editing and muscle movement, from one of its deriviatives without de novo synthesis.

"Hypoxanthine riboside salvage" was introduced in this GO term as a new synonym since the original definition incorrectly implied that "inosine" and "hypoxanthine riboside" are two different substances. Further, this revised definition is of benefit both to domain experts, and to biologists of other specializations, who will understand at a glance the physiological role of a gene annotated with this term.

## Conclusion

The methods introduced in this paper offer what we believe to be a reliable means for assessing the quality of terms and their definitions in ontologies and taxonomies. By using these methods to rank GO definitions and terms, we have demonstrated their suitability in assisting ontology curators by drawing their attention to ill-defined terms. The fact, revealed by our ontology alignment, that other ontologies suffer shortcomings similar to if not worse than GO's, leads us to conclude that improving definitions in GO and in other terminologies is more than a matter of importing definitions from one ontology to another and will instead require a good deal of manual curation. However, once problematic terms have been located by the methods introduced in this paper, text mining approaches as those described in [39-43], can be used to help ontology curators in the goals of maximizing intelligibility and avoiding circularity and thereby in increasing the utility of the ontology as a whole.

## Methods

For the calculation of the indexes, definitions and the names of terms had to be tokenised, i.e. word boundaries had to be defined. For this purpose we used white-space characters (blank and tab), punctuation marks and hyphens. Other tokenisers like certain special characters may well be used for other purposes.

Our methods are outlined in the workflow in Figure 1. Our first step was accordingly to identify terms that have no definition at all, which were irrelevant to the first steps of the analysis. Of the remainder, we first identified those terms whose definitions possess a high degree of circularity. We then scored the intelligibility of the definitions of the remaining terms. These steps resulted in a list of terms which are either undefined or whose definitions are marked by low intelligibility high circularity. It could however be argued that if a term (or one of its synonyms) is intelligible in its own right, then the term itself can serve as its own definition. Thus, we used the intelligibility of the names and synonyms of the terms to narrow down the list of problematic terms.

We then explored to what extent GO's problematic definitions can be improved by borrowing definitions from other ontologies. Our automated methods for mapping, outlined in [44], are designed to align equivalent terms and achieve a precision of >0.95 (i.e. >95% of all mappings are correct in the sense that they coincide with preliminary evaluations carried out manually).

We thus aligned GO pairwise to ontologies and controlled vocabularies such as MeSH [37], WordNet 2.0 [36], and the Enzyme Nomenclature [38]. In addition, we used 3,371 manual mappings between GO and the Enzyme Nomenclature [45]. We also used the mappings between the Enzyme Nomenclature and MeSH, which are included in MeSH itself. We found a total of 14,495 mappings between terms from these 4 ontologies, out of which 5,284 link GO terms to MeSH, WordNet or the Enzyme Nomenclature. In these other ontologies (EC, MeSH, WordNet) we found counterparts to 2,831 ill defined GO terms.

All computations were carried out on the basis of GO's February 2004 release, within the ONDEX framework [44,46], which is a system for automated ontology alignment, ontology-based text indexing and database integration. A separate publication on the ontology alignment methods is currently in preparation. In order to keep the methods and computations of the workflow generic (so that they can be applied also to other controlled vocabularies), we treated all GO terms in the same way, i.e. we did not differentiate between "unlocalized terms", "obsolete terms" or other GO particularities such as its terms for enzymatic functions (as discussed above in section "Intelligibility of definitions of GO terms"). This should not, however, have significantly influenced the results, since GO has classified only 794 out of its 17,110 terms as obsolete. Our results still include the information from GO whether a term is obsolete. Those who wish to use these results as the basis for further improvements in GO can thus easily filter out the corresponding expressions.

## Authors' contributions

JK drafted the manuscript. KM and BS contributed the principles that led to the development of the circularity and intelligibility indexes and participated in the preparation of the manuscript. AR, AS and JK developed, implemented and applied the computational methods. All authors read and approved the final manuscript.

## Supplementary Material

Additional File 1 Circular definitions, circularity index ≥ 0.5 (See also Figure 1, A1) Click here for file

Additional File 2 Non-circular definitions, circularity index < 0.5 (See also Figure 1, A2) Click here for file

Additional File 3 Unintelligible definitions, intelligibility index < 0.7 (See also Figure 1, B1) Click here for file

Additional File 4 Intelligible definitions, intelligibility index ≥ 0.7, (See also Figure 1, B2) Click here for file

Additional File 5 Unintelligible terms, intelligibility index < 0.7 (See also Figure 1, C1) Click here for file

Additional File 6 Unintelligible terms with proposed alternative definitions, intelligibility index < 0.7 (See also Figure 1, C1) Click here for file

Additional File 7 Intelligible terms, intelligibility index ≥ 0.7, (See also Figure 1, C2) Click here for file

Additional File 8 Evaluation of the circularity index, full evaluation results for the intelligibility score and the circularity index Click here for file

Additional File 9 Evaluation of the intelligibility index, full evaluation results for the intelligibility score and the intelligibility index Click here for file

## Figures and Tables

**Table 1:** Evaluation results for the intelligibility score and the circularity score. Subjects were asked to rate the circularity and intelligibility of definitions by answering 3 questions. The top and low scoring GO-terms were presented in random order and the score was not visible to the evaluators. Explanation on how to read the results: Q1 – Biol. 1 disagreed with only 6/50 terms that received a high circularity index, whereas he agreed with 49/50 terms, that received a low circularity score. Q2: Biol. 1 classified 49/50 terms that received a high intelligibility and 44/50 terms with a low intelligible index, as "roughly intelligible". Q3: Biol. 1 classified 29/50 terms that received a high intelligibility and 3/50 terms with a low intelligible index, as "fully intelligible".

	Q1: Is the definition not circular, i.e. does the definition provide more information than the term itself?		Q2: Is the definition intelligible, i.e. did you roughly understand the meaning of the GO entry by reading the definition?		Q3: Is the definition intelligible, i.e. are you able to fully understand the meaning of the GO entry without requiring further reading of other sources?	
Person score	Circular>	Not Circular	Intelligible	Not Intelligible	Intelligible	Not Intelligible

Biol. 1 – PostDoc	6/50	49/50	49/50	44/50	29/50	3/50
Biol. 2 – PostDoc	1/50	48/50	50/50	22/50	40/50	1/50
Biol. 3 – BSc	1/50	46/50	44/50	49/50	45/50	2/50
Bioinf. – MSc	3/50	46/50	45/50	4/50	48/50	1/50
